# Patients with mosaic methylation patterns of the Prader-Willi/Angelman Syndrome critical region exhibit AS-like phenotypes with some PWS features

**DOI:** 10.1186/s13039-016-0233-0

**Published:** 2016-03-22

**Authors:** Umut Aypar, Nicole L. Hoppman, Erik C. Thorland, D. Brian Dawson

**Affiliations:** Department of Laboratory Medicine and Pathology, Mayo Clinic, 200 First Street SW, Rochester, MN 55905 USA

**Keywords:** 15q11.2-q13, PWASCR, Chromosomal microarray, MS-MLPA, Mosaic methylation

## Abstract

**Background:**

Loss of expression of imprinted genes in the 15q11.2-q13 region is known to cause either Prader-Willi syndrome (PWS) or Angelman syndrome (AS), depending on the parent of origin. In some patients (1 % in PWS and 2–4 % in AS), the disease is due to aberrant imprinting or gene silencing, or both.

**Results:**

We report here a 4-year-old boy on whom a chromosomal microarray (CMA) was performed due to mild hand tremors, mild developmental delays, and clumsiness. CMA revealed absence of heterozygosity (AOH) spanning the entire chromosome 15, suggesting uniparental isodisomy 15. The patient had no definitive phenotypic features of PWS or AS. Methylation-sensitive multiplex ligation-dependent probe amplification (MS-MLPA) was performed to determine the parent of origin of the uniparental disomy (UPD) by examining methylation status at maternally imprinted sites. Interestingly, our patient had a mosaic methylation pattern. We identified nine additional previously tested patients with a similar mosaic methylation pattern. CMA was performed on these individuals retrospectively to test whether patients with mosaic methylation are more likely to have UPD of chromosome 15. Of the nine patients, only one had regions of AOH on chromosome 15; however, this patient had numerous regions of AOH on multiple chromosomes suggestive of consanguinity.

**Conclusion:**

The patients with mosaic methylation had milder or atypical features of AS, and the majority also had some features characteristic of PWS. We suggest that quantitative methylation analysis be performed for cases of atypical PWS or AS. It is also important to follow up with methylation testing when whole-chromosome isodisomy is detected.

## Background

Prader-Willi syndrome (PWS) and Angelman syndrome (AS) are distinct disorders caused by lack of expression of paternally (PWS) or maternally (AS) imprinted genes at 15q11–15q13, termed the Prader-Willi/Angelman syndrome critical region (PWASCR). Approximately 99 % of PWS and 80 % of AS cases are caused by large deletions, uniparental disomy (UPD) for chromosome 15, or imprinting center defects, respectively, while approximately 10 % of AS is due to point mutations in *UBE3A* [1–3]. In some patients (1 % in PWS and 2–4 % in AS), the disease is due to aberrant imprinting or gene silencing, or both [4–7]. While these disorders are an outcome of aberrations of the same region, the phenotypes are strikingly different. PWS is characterized by hypotonia, initial growth failure followed by obesity, hyperphagia, intellectual disability, developmental delay, and hypogonadism [8]. AS is characterized by microcephaly, severe intellectual disability, profound speech deficits, gait ataxia, seizures, and happy disposition [3].

Despite these differences in phenotype, patients with a combination of these features have been described. Some of these patients were found to have a mosaic methylation pattern of the PWASCR. To further understand the mechanism of pathogenesis and phenotype of patients with mosaic methylation pattern, we studied 10 patients with mosaic methylation pattern tested between June 2007 and June 2013 at the Mayo Clinic Clinical Molecular Genetics and Clinical Cytogenetics laboratories.

## Results

**Fig. 1:**
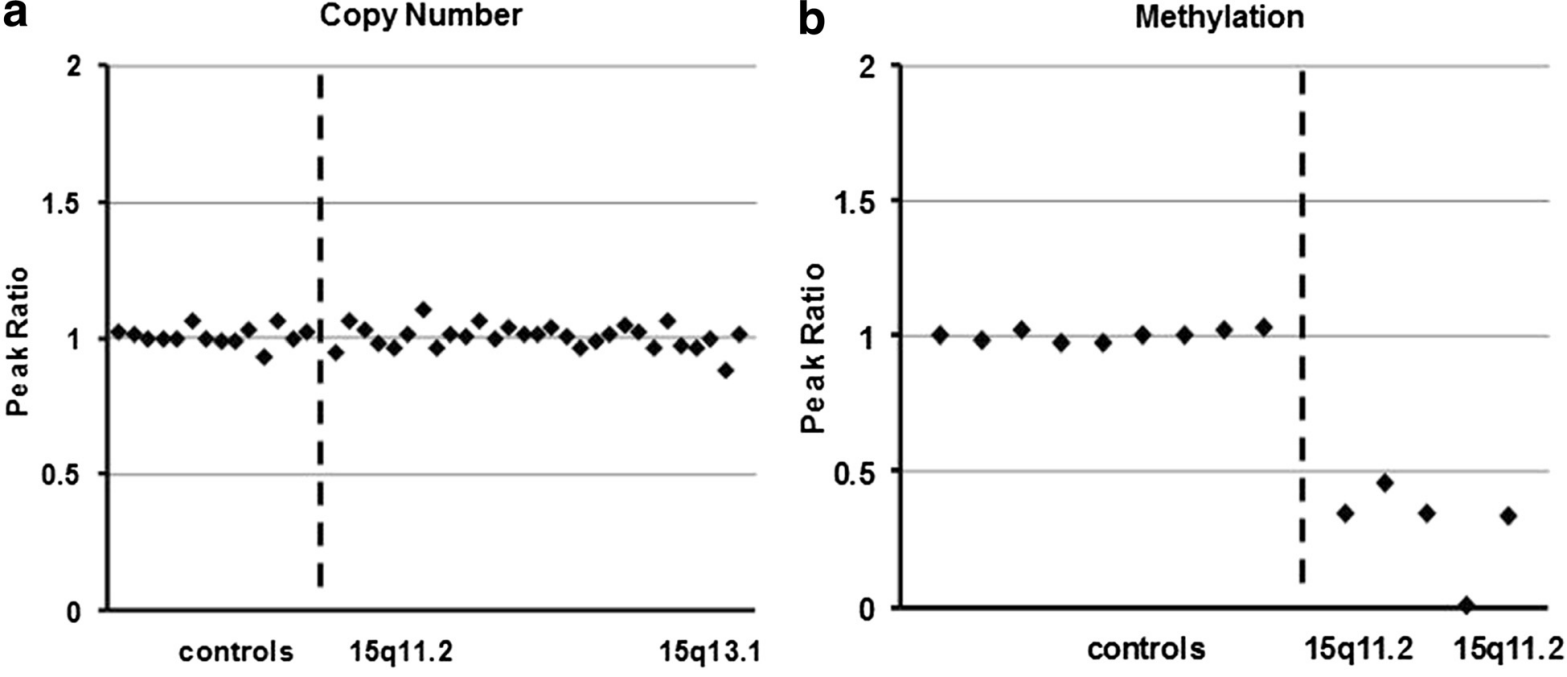
Results of Methylation-Sensitive Multiplex Ligation-Dependent Probe Amplification in Cases With Mosaic Methylation Pattern of the Prader-Willi/Angelman Syndrome Critical Region. Copy number peak ratios are determined by comparing patients with normal controls (2 copies/2 copies = 1.0), in this case no deletion is observed (**a**). The methylation probes are designed to hybridize to maternally imprinted loci; therefore, when compared to normal controls, in the absence of a deletion, patients with Angelman syndrome are expected to have no methylation (plotting to zero), while patients with Prader-Willi syndrome should have 2 methylated copies (ratio of 2). Interestingly, our patient (case 1) had a ratio slightly below 0.5 on average (**b**)

**Fig. 2:**
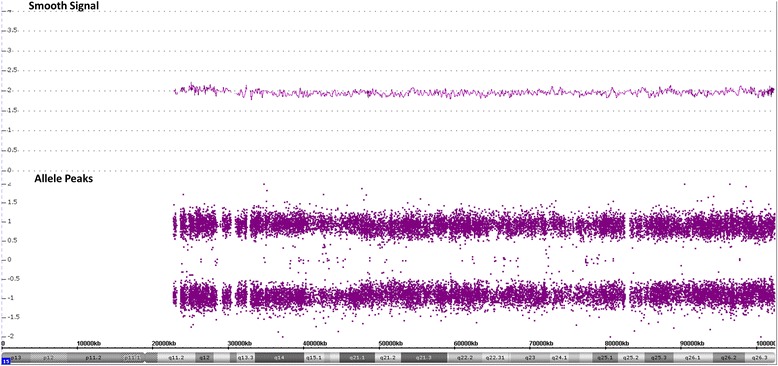
Results of Chromosomal Microarray in Case 1. Smooth signal is plotted at 2, indicating that two copies of the chromosome 15q arm are present without any deletion or duplication of the Prader-Willi/Angelman syndrome critical region. Allele peaks show the genotype calls. Genotype calls and allele dosage normalization are performed as follows: the formula for allele peaks is A – B, where A is the signal of the A allele and B is the signal of the B allele. The allele peaks are normalized such that AA = 1, AB = 0, and BB = −1. Therefore, the absence of heterozygosity would be observed as loss of the AB allele peaks (plotted at 0) with only AA (plotted at 1) and BB (plotted at −1) allele peaks present, as shown with case 1

To date, we have identified 10 cases with mosaic methylation pattern of the PWASCR tested at the Mayo Clinic Clinical Molecular Genetics and Cytogenetics laboratories (Table 1). The specimens were all from younger patients, ranging from 2 to 11 years old, and both sexes were equally represented (6 males, 5 females). Figure 1a and 1b show representative normal copy number and mosaic methylation pattern, respectively, of the PWASCR as detected by MS-MLPA (case 1). All of the cases except cases 1 and 2 had normal copy number and heterozygosity of chromosome 15 as detected by CMA. Cases 1 and 2 had normal copy number of chromosome 15; however, absence of heterozygosity (AOH) was identified. AOH spanning the entire chromosome 15, suggesting uniparental isodisomy 15, was observed for case 1 (Fig. 2a), and case 2 had regions of AOH on chromosome 15; however, this case had numerous regions of AOH on multiple chromosomes, suggesting consanguinity.

**Table 1:** Cases With Mosaic Methylation Pattern of the Prader-Willi/Angelman Syndrome Critical Region, Including Age, Sex, MS-MLPA Results, CMA for Chromosome 15 Results, and Phenotype

Patient	Age (y)	Sex	MS-MLPA	CMA, Chromosome 15	Angelman Syndrome Features					Prader-Willi Syndrome Features				
					Microcephaly	Speech Delay	Gait Ataxia	Seizures	Happy Disposition	Hypotonia	Growth Failure	Obesity	Hyperphagia	Hypogonadism
1	4	M	Mosaicism for maternal copy	Hmz 15	--	--	Y	N	--	--	--	N	--	--
2	11	F	Mosaicism for maternal copy	Partial hmz 15	--	Y	--	--	--	Y	--	--	--	--
3	3	M	Mosaicism for maternal copy	Normal	Y	Y	Y	Y	N	Y	N	N	N	N
4	6	F	Mosaicism for maternal copy	Normal	N	Y	N	N	Y	Y	N	N	N	N
5	6	F	Mosaicism for maternal copy	Normal	--	--	--	Y	Y	--	--	--	--	--
6	6	M	Mosaicism for maternal copy	Normal	--	Y	--	N	--	--	--	Y	Y	--
7	2	F	Mosaicism for maternal copy	Normal	--	--	--	--	--	--	--	--	--	--
8	8	M	Mosaicism for maternal copy	Normal	--	--	--	--	--	--	--	--	--	--
9	9	M	Mosaicism for maternal copy	Normal	--	--	--	--	--	--	--	--	--	--
10	10	M	Mosaicism for maternal copy	Normal	Y	Y	Y	--	Y	--	--	N	Y	--

Abbreviations: *CMA* chromosomal microarray, *F* female, *hmz* homozygous, *M* male, *MS-MLPA* methylation-sensitive multiplex ligation-dependent probe amplification, *N* not present, *Y* present, −-, information not available

Case 1 was a 4-year-old boy with mild developmental delays, mild hand tremors, and clumsiness (ataxia). Results of fragile X testing were negative. CMA identified nonmosaic AOH spanning the entire chromosome 15, suggestive of uniparental isodisomy 15 (Fig. 2a). The patient had no phenotypic features definitively suggestive of PWS or AS. MS-MLPA was performed to determine the parent of origin of the UPD by examining methylation status at maternally imprinted sites. When compared to normal controls, in the absence of a deletion, patients with AS are expected to have maternally imprinted sites with no methylation (plotting to zero), while patients with PWS are expected to have sites with 2 methylated copies (ratio of 2). Interestingly, our patient had a ratio of ~0.3 (Fig. 1b). After the test results were received, additional clinical information was obtained, which showed that the patient was much less severely affected than expected for AS. While he had features similar to those of AS, such as essential tremors and clumsiness (ataxia), he had no seizures, dysmorphic features, or obesity.

We then identified nine additional previously tested patients with a similar mosaic methylation pattern. CMA was performed to test whether patients with mosaic methylation are more likely to have UPD of chromosome 15. Of the nine patients, only one (case 2) had regions of nonmosaic AOH on chromosome 15; however, this patient had numerous regions of AOH on multiple chromosomes suggestive of consanguinity. Case 2 was an 11-year-old girl with developmental delay, speech delay, and hypotonia. She also had normal results of chromosome, PWAS fluorescence in situ hybridization (FISH), and UPD studies.

The remaining eight cases had no abnormalities of chromosome 15 or any other pathogenic copy number variations. Case 3 was a 3-year-old boy who had an atypical or milder AS phenotype, including speech delay with apraxia, global developmental delay, mild hypotonia, microcephaly (head circumference at second percentile), and abnormal electroencephalogram showing nocturnal seizures. The patient was also born with congenital heart disease, but there were no other health concerns (weight at 96th percentile, height at 94th percentile). Results of laboratory tests, including chromosomes, fragile X, UPD, and *UBE3A* sequencing, were all normal. At about age 7 years, the patient was also noted to have mild to moderate intellectual disability, attention-deficit/hyperactivity disorder, behavioral issues, sleep-related problems, and extremely reduced motor coordination (ataxia).

Case 4 was a 6-year-old girl with a happy disposition, developmental delay, hypotonia, and speech delay with apraxia. However, she progressed well cognitively and performed on track with peers academically. There were no other health concerns, and results of laboratory tests, including metabolic, mitochondrial DNA, and muscle biopsy, were normal.

Case 5 was a 6-year-old girl who had milder features of AS. She had a happy demeanor, developmental delay, intellectual disability, seizures, macrocephaly, and diabetes.

Case 6 was a 6-year-old boy who was obese partly due to his constant obsession to hide, hoard, and sneak food (hyperphagia). The patient had developmental delay, speech delay (nonverbal), autism, and temper/aggression issues but no seizures. He did not have an obvious movement disorder but received physical and occupational therapy earlier in life. Results of chromosome, PWAS FISH, and fragile X tests were all normal.

Case 7 was a 2-year-old girl with developmental delay, mild hypertonia, reflux, and weight loss due to pancreatic insufficiency.

Case 10 was a 10-year-old boy who was initially thought to have PWS because of his moderate intellectual disability, developmental delay, skin picking, and hyperphagia. However, he also had AS features, including microcephaly (head circumference at 25th percentile), absent speech, excitability, happy demeanor, ataxic gait, insomnia, abnormal eye movement, strabismus, umbilical hernia, and pectus excavatum. At age 12, he had failure to thrive (weight at 10th percentile), short stature (height at 50th percentile), and early puberty.

For cases 8 and 9, no clinical information was available.

## Discussion

Although PWS and AS are caused by lack of paternal or maternal, respectively, expression of imprinted genes at the same region (PWASCR), they are phenotypically distinct disorders. PWS is characterized by hypotonia, initial growth failure followed by obesity, hypogonadism, and variable degrees of intellectual disability. Typical features of classic AS are severe intellectual disability, profound language deficits, gait ataxia, seizures, microcephaly, and happy demeanor. In contrast to these distinct features, patients with a combination of these phenotypes have been described in the literature.

In 1999, Gillessen-Kaesbach et al. reported seven atypical AS patients with obesity, hypotonia, and intellectual disability who were initially thought to have PWS [9]. These patients also lacked ataxia, microcephaly, and AS facial features, and three of the seven had active speech. However, their methylation pattern was suggestive of AS. At least five of these seven patients had a weak maternal band indicative of mosaicism. Buiting et al. described six additional patients with atypical AS, but also ten patients with typical symptoms of AS who had a mosaic methylation pattern [5]. Their study found that the patients with methylation mosaicism had a broad clinical spectrum that ranged from typical AS, through mild AS, to atypical AS. Methylation mosaicism was rare in their patients with PWS; however, two of the patients with PWS also showed mosaicism. Their study concluded that the epimutations of the maternal chromosome are often mosaic. Nazlican et al. reported 24 patients with mosaic imprinting defects and also concluded that these patients can have milder or atypical AS [10]. In their study, patients with milder AS had a higher percentage of normal cells; however, patients with the same ratio of normal and imprinting defect cells did express different phenotypes. In contrast, Wey et al. described a patient with mosaic imprinting defect who had relatively high levels of normally methylated cells, and yet this patient had an almost typical phenotypic expression of PWS [11]. This highlights the difficulty in assigning severity and the spectrum of phenotypic expression on the basis of the ratio of normal and abnormal cells. Lawson-Yuen et al. reported two cases with atypical AS, one of whom had a mosaic methylation pattern [12]. They concluded that the diagnosis of AS should not be ruled out in patients with milder presentations, such as those with some language development and normocephaly. Camprubi et al. identified two patients with AS who had mosaic methylation patterns and, interestingly, one of these patients displayed a mild AS phenotype while the other displayed a PWS-like phenotype [13].

The phenotypes associated with mosaic methylation pattern of the PWASCR in our patient cohort are in accordance with those previously reported. All of the patients had mosaic methylation pattern of the maternally imprinted genes, suggesting a diagnosis of AS spectrum. While all the patients had milder or atypical features of AS (Table 1), the majority (5 of 8 cases with clinical information) had some features characteristic of PWS, such as hypotonia (cases 2, 3, and 4), hyperphagia (cases 6 and 10), and obesity (case 6). Of note, most of the cases had limited clinical information available; therefore, the presence of PWS-like features in the remainder of patients cannot be ruled out.

The mechanism of pathogenesis for case 1 is especially interesting. There are several reports of mosaic UPD as a consequence of trisomy 15 rescue that resulted in PWS [14–16], and recently, Izumi et al. reported 2 cases with mosaic UPD that resulted in mosaic methylation pattern of the PWASCR [17]. In addition, patients with AOH on 15q11.2 have been described with features including developmental delay, intellectual disability, autism, epilepsy, feeding difficulty, and other features [18]. However, these cases are unlike case 1 presented in this study. For case 1, the UPD detected by CMA is not mosaic, while the methylation pattern detected by MS-MLPA is mosaic for the maternally imprinted genes. Also, the patients with mosaic UPD tend to have a classic PWS phenotype, while our case 1 has atypical AS.

## Conclusions

On the basis of our data, we conclude that patients with mosaic methylation pattern of the maternally imprinted genes tend to have milder or atypical AS and the majority have features that resemble PWS. We recommend that quantitative methylation analysis be performed for cases of atypical PWS or AS. It is also important to follow up with methylation testing when whole-chromosome isodisomy is detected. However, the mechanism behind methylation mosaicism remains unclear.

## Methods

### Cases

Case 1 was initially detected by chromosomal microarray (CMA) performed at the Mayo Clinic Clinical Cytogenetics Laboratory, and methylation-sensitive multiplex ligation-dependent probe amplification (MS-MLPA; MRC-Holland) was performed subsequently. After case 1 was identified, an additional 9 cases with similar mosaic methylation pattern of the PWASCR as detected by MS-MLPA were identified through the results database of the Mayo Clinic Clinical Molecular Genetics Laboratory. In these 9 cases, CMA was performed as part of this study. For almost all the cases, limited phenotypic information was available because the only sources were the reason for ordering the test and any information provided by the ordering physician by phone when testing was done.

### DNA extraction and MS-MLPA

Genomic DNA extraction was performed using the Gentra Puregene method (Qiagen) on total blood leukocytes. MS-MLPA was performed using Salsa MS-MLPA Kit ME028 (MRC-Holland). Briefly, 200 ng of genomic DNA was denatured at 98 °C for 10 min. Probes were then hybridized at 60 °C for 16 to 24 h. On completion, each reaction was divided into 2 tubes of equal volume; the ligation reaction alone was performed in 1 tube while ligation and simultaneous digestion with *Hha*I were performed in the second, both at 49 °C for 30 min. Each reaction then underwent polymerase chain reaction amplification using universal primers according to the manufacturer’s protocol. On completion, 8.5 μL Hi-Di Formamide (Life Technologies) and 0.5 μL GeneScan 500 ROX Size Standard (Life Technologies) were added to 1 μL of each reaction. This mixture was denatured at 95 °C for 5 min and placed on an ice-water slurry for an additional 5 min. Samples were then subjected to capillary electrophoresis on an ABI 3100 (Applied Biosystems). MS-MLPA results were analyzed using GeneMarker version 1.8 (SoftGenetics, LLC) to determine copy number and methylation status of the PWASCR [19]. The fluorescent signals from the copy number probes are compared to normal controls. The methylation probes are designed to interrogate maternally imprinted loci (maternal allele methylated). Therefore, when compared to normal controls, the ratio of methylated probes will increase accordingly in the presence of additional copies of the maternal allele but not because of the presence of additional paternal alleles. When compared to normal controls, in the absence of a deletion, patients with AS are expected to have maternally imprinted sites with no methylation (plotting to zero), while patients with PWS are expected to have sites with 2 methylated copies (ratio of 2).

### Chromosomal microarray

CMA was performed using CytoScan HD (Affymetrix) according to the manufacturer’s protocol. Data were analyzed using Chromosome Analysis Suite (ChAS) software (Affymetrix).

## Consent

Mayo Clinic IRB approval was obtained (ID: 13–009236).
